# Establishment of Human Pluripotent Stem Cell‐Derived Skin Organoids Enabled Pathophysiological Model of SARS‐CoV‐2 Infection

**DOI:** 10.1002/advs.202104192

**Published:** 2021-12-31

**Authors:** Jie Ma, Jia liu, Dunqin Gao, Xiao Li, Qiyu Zhang, Luye Lv, Yujie Wang, Jun Li, Yunping Zhu, Zhihong Wu, Hengrui Hu, Yufeng Li, Longda Ma, Qian Liu, Zhihong Hu, Shuyang Zhang, Yiwu Zhou, Manli Wang, Ling Leng

**Affiliations:** ^1^ State Key Laboratory of Proteomics Beijing Proteome Research Center National Center for Protein Sciences (Beijing) Beijing Institute of Lifeomics Beijing 102206 China; ^2^ State Key Laboratory of Virology Wuhan Institute of Virology Center for Biosafety Mega‐Science Chinese Academy of Sciences Wuhan 430071 China; ^3^ Stem cell and Regenerative Medicine Lab Department of Medical Science Research Center State Key Laboratory of Complex Severe and Rare Diseases Translational Medicine Center Peking Union Medical College Hospital Chinese Academy of Medical Sciences and Peking Union Medical College Beijing 100730 China; ^4^ Department of Dermatology and Venereology Peking Union Medical College Hospital Chinese Academy of Medical Sciences and Peking Union Medical College Beijing 100730 China; ^5^ Institute of NBC Defense Beijing 102205 China; ^6^ Basic Medical School Anhui Medical University Anhui 230032 China; ^7^ Department of Forensic Medicine Tongji Medical College of Huazhong University of Science and Technology Wuhan 430010 China; ^8^ Department of Cardiology Peking Union Medical College Hospital Chinese Academy of Medical Sciences and Peking Union Medical College Beijing 100730 China

**Keywords:** COVID‐19, hair follicle, nervous system, proteomics, SARS‐CoV‐2, skin organoids

## Abstract

Coronavirus disease 2019 (COVID‐19) patients with impact on skin and hair loss are reported. Severe acute respiratory syndrome coronavirus 2 (SARS‐CoV‐2) is detected in the skin of some patients; however, the detailed pathological features of skin tissues from patients infected with SARS‐CoV‐2 at a molecular level are limited. Especially, the ability of SARS‐CoV‐2 to infect skin cells and impact their function is not well understood. A proteome map of COVID‐19 skin is established here and the susceptibility of human‐induced pluripotent stem cell (hiPSC)‐derived skin organoids with hair follicles and nervous system is investigated, to SARS‐CoV‐2 infection. It is shown that KRT17+ hair follicles can be infected by SARS‐CoV‐2 and are associated with the impaired development of hair follicles and epidermis. Different types of nervous system cells are also found to be infected, which can lead to neuron death. Findings from the present work provide evidence for the association between COVID‐19 and hair loss. hiPSC‐derived skin organoids are also presented as an experimental model which can be used to investigate the susceptibility of skin cells to SARS‐CoV‐2 infection and can help identify various pathological mechanisms and drug screening strategies.

## Graphical abstract

A proteome map of coronavirus disease 2019 skin is established. A human‐induced pluripotent stem cell‐derived skin organoids model is constructed and used to show that KRT17+ hair follicles and different types of nerve cells can be infected by severe acute respiratory syndrome coronavirus 2 and are associated with the impaired development of hair follicles and epidermis.

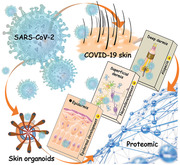

## 1 Introduction

The severe acute respiratory syndrome coronavirus 2 (SARS‐CoV‐2) infection affects multiple organs such as the lungs, gut, kidney, brain, and skin.^[^ ^1^, ^2^, ^3^, ^4^ ^]^ Previous reports have shown that coronavirus disease 2019 (COVID‐19) patients developed acral areas of erythema with vesicles or pustules (pseudochilblain), acral areas with erythematous rash, widespread urticaria, chickenpox‐like vesicles, and maculopapular eruptions.^[^ ^5^, ^6^ ^]^ In addition, it has been reported that depending on the clinical severity of the disease COVID‐19 patients experienced hair loss on their scalp.^[^ ^7^ ^]^ However, there is no evidence yet about any abnormality observed in the hair follicles of the COVID‐19 patients.

Epidermal stem cells (EpSCs) of the basal layer of skin comprise of two types of cells including interfollicular epidermal stem cells and hair follicle stem cells (HFSCs), which maintain homeostasis and wound healing in the skin. EpSCs have undifferentiated proliferative progenitor cells expressing keratins, including keratin 5 (K5) and keratin 14 (K14).^[^ ^8^ ^]^ These progenitors not only replenish the basal layer through self‐renewal, but also progressively migrate upward through the epidermis, differentiating to form mature keratinocytes expressing keratin 1 (K1), keratin 10 (K10), and involucrin, and finally the outer layers of the terminally differentiated, dead stratum corneum cells.^[^ ^9^ ^]^ EpSCs can be linked to the adjacent basement membrane (BM) zone through integrin *α*3*β*1‐rich focal adhesions and hemidesmosome complex including type IV collagens, nidogens, laminins, etc. to maintain their proliferative capacity and their ability to migrate in response to injury.^[^ ^10^ ^]^ In addition, cell‐cell interaction associated with proteins including E‐cadherin (ECAD) and desmosome can coordinate cytoskeleton dynamics and directed movements. Loss of these intercellular and cell‐matrix junctions could lead to the impairment of the microenvironment and the mechanical strength of EpSCs. During hair follicle formation, the epidermis first forms placodes with dermal condensates. Then, cells of the placode form hair germs, and the matrix cells encapsulate the dermal papilla (DP). The matrix produces a three‐layered inner root sheath (IRS) and the outer root sheath (ORS), whose cells contact the BM zone directly.^[^ ^10^ ^]^ Wnt/*β*‐catenin signaling is crucial for the maintenance of interfollicular epidermal stem cells along with self‐renewal and formation of hair follicles.^[^ ^11^ ^]^ Also, the Wnt signal is involved in the central nervous system, directing progenitor neuronal cells into neuronal characteristics.^[^ ^12^ ^]^ Interestingly, it has been reported that hair follicles are the important target tissues for classical neurohormones, neurotrophins, and neuropeptides, signifying the communication between hair follicles and the nervous system.^[^ ^13^ ^]^

Organoids displaying key structural and functional features of natural tissues and organs can be grown, mimic the physiological characteristics of tissue growth and wound repair. Therefore, organoids are widely used for the investigation of mechanisms of tissue regeneration, disease model, and drug screening.^[^ ^14^ ^]^ Several kinds of organ models have been established, such as intestine,^[^ ^15^ ^]^ brain,^[^ ^16^ ^]^ kidney,^[^ ^17^ ^]^ lung,^[^ ^18^ ^]^ liver,^[^ ^19^ ^]^ pancreas,^[^ ^20^ ^]^ heart,^[^ ^21^ ^]^ retina,^[^ ^22^ ^]^ and skin.^[^ ^23^ ^]^ During the COVID‐19 pandemic, multiple human pluripotent stem cell (hiPSC)‐derived organoids have been used to model SARS‐CoV‐2 infection in many organs.^[^ ^24^, ^25^, ^26^, ^27^, ^28^ ^]^ This powerful and revolutionary technology has promoted the emergence of new biomedical models, as well as the research and treatment of various endogenous and exogenous injuries or infectious diseases in human beings. However, so far, there has been no report about the use of 3D skin models for studying the pathological mechanisms in SARS‐CoV‐2 infected skin tissues. In the present work, we have established a hiPSC‐derived skin organoid with hair follicles and nervous system, which is sensitive to external damage. The skin organoid was used to investigate the cell types affected by SARS‐CoV‐2 infection and the pathological characteristics associated with a skin infection. Based on the results, we present evidence of the functional consequences of skin infected by SARS‐CoV‐2 at cellular and molecular levels.

## 2 Results

### 2.1 Comprehensive Proteomics Landscape of Clinical COVID‐19 Skin Samples

**Figure 1:**
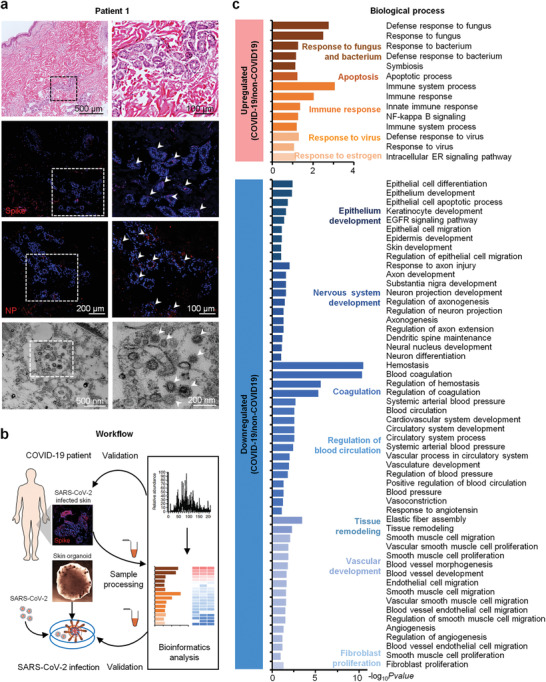
Quantitative proteome profiling of protein signatures in COVID‐19 skin tissues. a) H&E, spike, and NP proteins staining, and transmission electron microscope analysis of SARS‐CoV‐2 infected skin tissues (Scale bar: 200 and 100 µm, 500 and 200 nm). The white solid arrows indicate the SARS‐CoV‐2 proteins in COVID‐19 skin tissues. b) Schematic representation of the experimental workflow of the skin organoids culture, SARS‐CoV‐2 infection, quantitative proteomics, bioinformatics analysis, and biological validation. c) Biological process analyses of the upregulated and downregulated‐expressed proteins between COVID‐19 (*n* = 5) and control (*n* = 6) skin tissues. Upregulated‐expressed and downregulated‐expressed proteins: *t*‐test, Benjamini–Hochberg (BH) adjusted *p*‐value < 0.01 and log_2_COVID‐19/Control > 1, and BH adjusted *p*‐value < 0.01 and log_2_COVID‐19/Control < −1.

Previous studies have shown that the skin can be infected by SARS‐CoV‐2.^[^ ^29^ ^]^ Our results revealed that the lymphocyte infiltration was observed in COVID‐19 skin—particularly adjacent to the epidermis and accessory glands in the dermis (**Figure** **1**a and Figure S1a, Supporting Information). These infiltrating lymphocytes includes CD3+/CD8+ T cells and CD68+ macrophages but not CD4+ T cells, CD19+/CD20+ B cells or myeloperoxidase‐ positive neutrophils (Figure S1b, Supporting Information). In addition, capillary endothelial cells swell in dermis and organized microthrombus could be see occasionally (Figure S1a, Supporting Information). Furthermore, the staining of spike and nucleoprotein (NP) proteins, as well as the electron microscopy results confirmed the presence of SARS‐CoV‐2 particles in the COVID‐19 skin tissues (Figure 1a). An integrated quantitative proteomics approach was used to detect proteomic changes in the SARS‐CoV‐2 infected skin (Figure 1b). Principal component analysis (PCA) revealed that the proteins identified in the skin tissues from the patients with COVID‐19 and the controls formed independent clusters (Figure S1c, Supporting Information). Functional analysis showed that the proteins upregulated during SARS‐CoV‐2 skin infection were mainly enriched in the biological processes involved in the response to microorganisms (bacteria, fungi, and viruses), apoptosis, immune response, and estrogen associated signaling; while the downregulated proteins were related to regulation of blood circulation, tissue remodeling and the development of epidermis, nervous system, and blood vessels, as compared to the control (Figure 1c). In addition, the most downregulated proteins in the cellular components category were associated with extracellular matrix (ECM), focal adhesion, cell‐cell junction, myelin sheath, melanosome, and cytoskeleton (Figure S1d, Supporting Information). These results indicate that ECM microenvironment, epithelial development, nervous system, circulation system, as well as cell junctions in skin tissue might be affected in patients with COVID‐19.

### 2.2 Formation of Skin Organoids with Various hiPSC‐Derived Skin Cells

**Figure 2:**
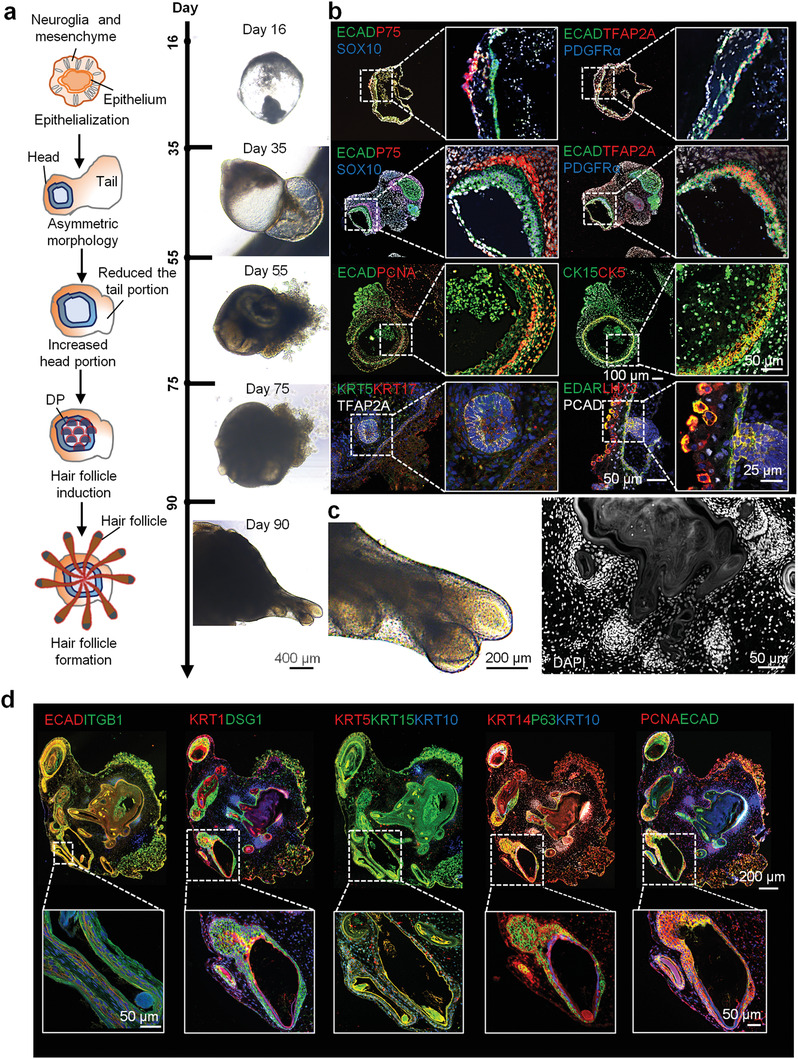
Formation of skin organoids in vitro. a) A schematic overview of the hiPSC‐skin organoid culture. The bright field representing organoids culture on days 16, 35, 55, 75, and 90 (Scale bar: 400 µm). b) Immunofluorescence of the epithelial cells markers (TFAP2A, ECAD, and KRT17) at day 15–35, mesenchyme‐associated marker (PDGFR*α*), neuroglia‐associated markers (SOX10 and P75), proliferation marker (PCNA), epithelial stem cells markers (KRT15 and KRT5) at day 55, epidermal germ cell markers (LHX2, PCAD, and EDAR), dermal condensates (PDGFR*α* and P75) at day 75. c) Bright field and immunofluorescence represent hair follicles of skin organoids at day 90 (Scale bar: 200 and 50 µm). d) Immunofluorescence of the epithelial cell marker (ECAD), epidermal basal cells markers (KRT15, KRT5, KRT14, and P63), mature epithelium markers (KRT1 and KRT10), proliferation marker (PCNA), desmosome (DSG1), and adhesive receptor (ITGB1) at day 120 (Scale bar: 200 and 50 µm).

Next, to study the different cell types involved in the COVID‐19 skin, a skin organoid model was constructed using hiPSCs and subjected to SARS‐CoV‐2 infection (**Figure** **2**a). To obtain uniform epithelial cysts, a stepwise differentiation of hiPSCs into ectoderm was achieved through the treatment with bone morphogenetic protein 4 (BMP4) and a TGF*β* inhibitor (SB431542) on Matrigel, following the previous study^[^ ^23^ ^]^ (Figure S2a, Supporting Information). From day 16 to 35, the organoids displayed TFAP2A+ ECAD+ epithelial cells and two subtypes of cranial neural crest (CNC)‐like cells, expressing mesenchyme‐associated marker, either platelet‐derived growth factor receptor alpha (PDGFR*α*) or the neuroglia‐associated markers (SOX10 and P75) (Figure 2a,b). Remarkably, by day 35, the organoids displayed asymmetric structures, which were divided into head and tail (Figure 2a). With continuous culture, the tail structure decreased gradually and would disappear in some organoids, and finally the skin organoids gained matured structure with different cell types. By day 55, the KRT15+ KRT5+ epithelial‐like stem cells stably expanded and the tail portion was reduced. Further, the organoids presented hair follicle structures, expressing the epidermal germ cell markers (LIM Homeobox Protein 2 [LHX2], P‐cadherin (PCAD), and ectodysplasin a receptor (EDAR)) and showed the presence of dermal condensates (SOX2, PDGFR*α*, and P75) by day 75 (Figure 2a,b), indicating that the hair follicle structures were formed during this period. Our previous study showed that TGF*β* induced protein (TGFBI) can enhance epidermal stem cell growth (Figure S2b, Supporting Information). After 15 d, the 3D/organoid cultures treated with TGFBI for a month, exhibited the typical stratified epithelium structure, with the expression of epidermal basal cell markers (P63, KRT14, and KRT5), mature epithelium markers (KRT10, KRT1, and involucrin), adhesive receptor (ITGB1) and desmosomes (DSG1 and DSG2) (Figure 2d and Figure S2c, Supporting Information). In addition, the organoids are also comprised of the components of the dermal‐epidermal junction zone, called basement membrane, which is the key niche of EpSCs, such as laminin, type IV collagen, and nidogen (Figure S2d, Supporting Information). Dermal components (Aggrecan and COL2A1) were also identified in these organoids (Figure S2e, Supporting Information). In addition, mature hair follicle structures were also formed in the skin organoids (Video S1, Supporting Information).

**Figure 3:**
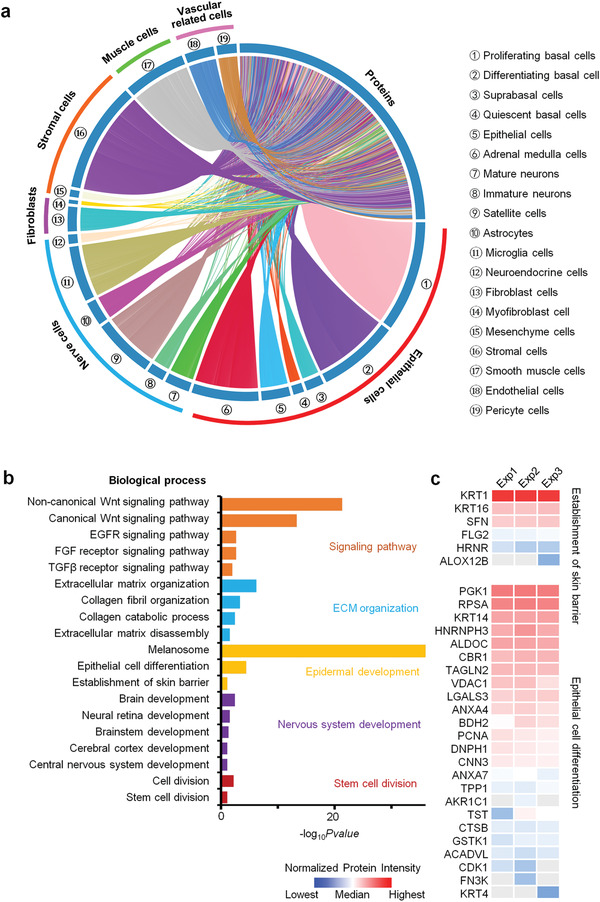
Quantitative proteome profiling of protein signatures in skin organoids. a) Proteins identified in the skin organoids (*n* = 3) were associated with different cell types according to the cell type signature gene sets in the Molecular Signatures Database (cell type enrichment *p*‐value < 0.001). b) Functional analysis of proteins identified in the control skin organoids. c) Heatmap analysis of the proteins identified in the control skin organoids that were associated with the establishment of the skin barrier and epithelial cell differentiation. Red and blue boxes indicate proteins with high and low intensities, respectively. Exp1, Exp2, and Exp 3 represent different biological repeat of the proteomics experiment (*n* = 3).

To further prove that the skin organoids resemble and mimic the physiological function of human skin tissue, we established the proteomic profile of mature skin organoids at day 140 after the culture was established (Table S1, Supporting Information). Results showed that the proteins identified in these organs were mainly enriched in epidermal cells, nerve cells, stromal cells, fibroblasts, muscle cells, and vascular‐related cells, after a comparison with the reported database (**Figure** **3**a). Biological process analysis showed that the proteins identified from skin organoids were mainly enriched in the processes of skin development including epidermal development, nervous system development, and stem cell division (Figure 3b). Several signaling pathways including canonical and noncanonical Wnt, epidermal growth factor receptor, fibroblast growth factor (FGF), and TGF*β* and TGFg associated with skin development were found to be enriched in the skin organoids. Especially, proteins associated with epidermal development that participated in the processes of establishment of the skin barrier (e.g., KRT1 and KRT16), epithelial cell differentiation (e.g., KRT14 and KRT4), and melanosome were identified in the skin organoids (Figure 3c and Figure S3a, Supporting Information). In addition, skeletal tissue associated processes including ECM and cytoskeleton organization and adhesion, as well as the lipid and energy metabolism associated processes were enriched in the skin organoids (Figure S3b, Supporting Information). 111 ECM proteins including core ECM proteins like 15 collagens, 31 glycoproteins, and 10 proteoglycans, as well as ECM associated proteins including 16 ECM regulators, 18 ECM‐affiliated proteins and 21 secreted factors, were identified (Figure S3c and Table S2, Supporting Information). These results indicate that we successfully constructed skin organoids with multiple structures and cellular functions.

### 2.3 SARS‐CoV‐2 Can Infect the Hair Follicle

**Figure 4:**
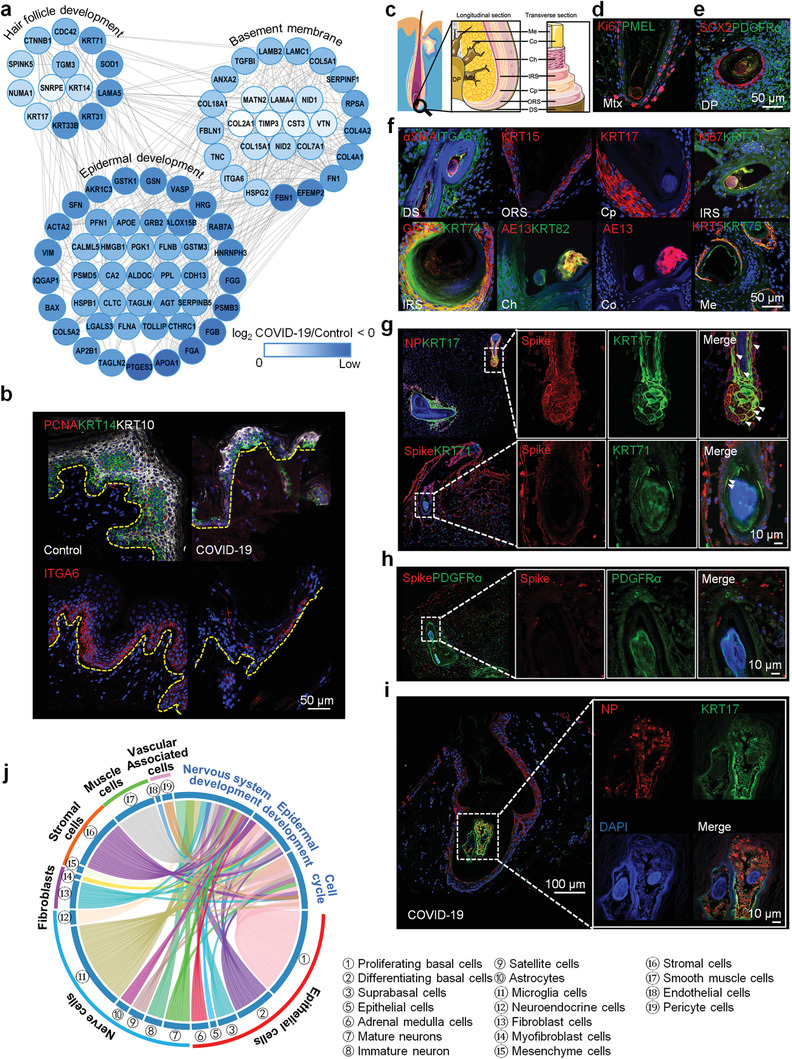
SARS‐CoV‐2 infects hair follicles. a) An interactome network of downregulated proteins from COVID‐19 skin tissues, when compared to the control group. b) Immunofluorescence of PCNA, KRT14, KRT10, and hemidesmosome complex (ITGA6) in the COVID‐19 and control skin tissues (Scale bar: 50 µm). c) A schematic of hair follicle construction. Immunofluorescence of hair follicles’ markers with different construction: d) Mtx (Ki67 and PMEL), e) DP (SOX2 and PDGF*α*), DS (*α*SMA and ITGA8), ORS (KRT15), Cp (KRT17), IRS (Ki67, KRT71, GATA3, and KRT74), Ch (AE13 and KRT82), Co (AE13), and Me (KRT5 and KRT75) (Scale bar: 50 µm). Mtx: matrix; DP: dermal papilla; DS: dermal sheath; ORS: outer root sheath; Cp: companion layer; IRS: inner root sheath; Ch: cuticle; Co: cortex; Me: medulla. f) In the normal organoids at day 140. Immunofluorescence of g) SARS‐CoV‐2 proteins (NP and spike), KRT71 and KRT71, h) spike and PDGF*α* in the SARS‐CoV‐2 infected organoids (Scale bar: 10 µm). i) Immunofluorescence of NP and KRT17 in the COVID‐19 skin tissues (Scale bar: 10 and 100 µm). j) Downregulated proteins between COVID‐19 (*n* = 3) and control skin tissues (*n* = 3) with functions of the nervous system, epidermal development, and cell cycle in the skin organoids were associated with different cell types according to the cell type signature gene sets in Molecular Signatures Database (cell type enrichment *p*‐value < 0.001). Downregulated‐expressed proteins: *t*‐test, BH adjusted *p*‐value < 0.01, and log_2_COVID‐19/Control < −1.

COVID‐19 patients are prone to hair loss.^[^ ^7^ ^]^ Further, we found that proteins associated with hair follicle development (KRT33B, KRT31, KRT71, KRT14, KRT17, CTNNB1, etc.) and epidermal development were significantly downregulated in the skin tissues of COVID‐19 patients (**Figure** **4**a). We previously found that BM structure is an important microenvironment supporting the fate and functional polarity of EpSCs.^[^ ^30^ ^]^ We observed that the proteins (type IV collagen, laminins, HSPG2, NID1, etc.) of BM structure and hemidesmosome complex (ITGA6) were severely damaged in the COVID‐19 skin tissues (Figure 4a). Immunofluorescence staining also showed that the expression of KRT14, KRT10, proliferating cell nuclear antigen (PCNA), P63, COL4A1, and ITGA6 downregulated in the COVID‐19 skin tissues or organoids, compared with the control group, which was consistent with the proteomics results (Figure 4b and Figure S4a, Supporting Information). These results suggest that the hair follicle development in COVID‐19 patients could be affected by SARS‐CoV‐2. To further examine if hair follicle might be one of the targets of SARS‐CoV‐2, we induced SARS‐CoV‐2 infection in these skin organoids.

**Figure 5:**
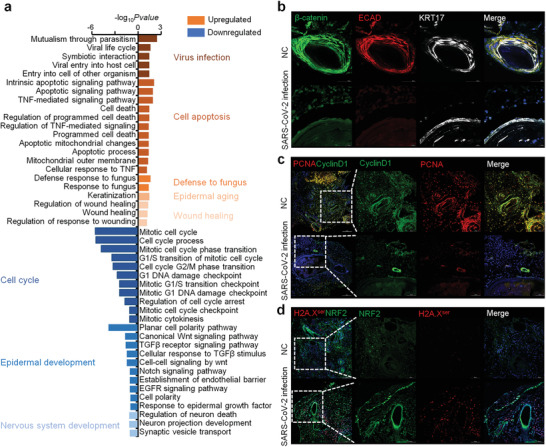
Pathological features of SARS‐CoV‐2 infected skin organoids. a) Functional analyses of the upregulated and downregulated‐expressed proteins identified in the SARS‐CoV‐2‐infected skin organoids compared with the controls. Upregulated‐expressed and downregulated‐expressed proteins: *t*‐test, BH adjusted *p*‐value <0.01 and log_2_Infected/Control >1, and BH adjusted *p*‐value < 0.01 and log_2_Infected/Control < −1. b) Immunofluorescence of *β*‐catenin, epithelial cell marker (ECAD) and KRT17, c) proliferation marker (PCNA) and Cyclin D1, and d) H2A.X^ser^ and NRF2 (Scale bar: 100 and 50 µm).

After the mature hair follicle structure was formed with the germ elongated into a peg‐like structure, in the skin organoids, Ki67+ epithelial cells were found to express premelanosome protein (PMEL) around the DP structure along with markers SOX2 and PDGFR*α* (Figure 4c–e). Further, we found that the hair follicles of skin organoids shared an outermost layer of alpha‐smooth muscle actin (*α*SMA) + integrin alpha 8 (ITGA8) + dermal sheath with characteristic arrector pili muscle. In addition, ORS markers (KRT15), ceruloplasmin markers (KRT17), IRS makers (Ki67, KRT71, GATA3, and KRT74), cuticle and cortex markers (AE13 and KRT82), and medulla markers (KRT5 and KRT75) of hair follicles were also found to be expressed in the skin organoids (Figure 4f). This indicates that all the hair follicle lineages formed from the outermost dermal sheath to the center of the medulla were well established in the organoids. The proteins associated with hair follicle development, hemidesmosome complex, desmosome, as well as BM were successfully identified in these skin organoids (Figure S4b, Supporting Information). Next, we exposed the mature organoids (day 140) to SARS‐CoV‐2. Testing the target structure of SARS‐CoV‐2 in these organoids revealed that SARS‐CoV‐2 could mostly target the KRT17+ hair follicle cells (Figure 4g and Figure S4c,d and Video S2, Supporting Information), however, less target the KRT71+ and PDGFR*α*+ hair follicle cells (Figure 4h). Pathological staining of the skin tissues infected by SARS‐CoV‐2 further proved that the virus could chiefly infect the KRT17+ hair follicle cells (Figure 4i and Figure S4e, Supporting Information). Besides, the downregulated proteins in the COVID‐19 skin tissues were mainly enriched in EpSCs, cell cycle, and epidermal development (Figures 4j and **5**a). The epithelization morphology of epidermal cells around KRT17 + hair follicles disappeared and the ability of cell proliferation decreased in the SARS‐CoV‐2 infected organoids, compared to the normal controls (Figure 5b,c). According to the pathological features, it is found that the cell cycle of infected organoids could be caused by DNA damage after SARS‐CoV‐2 infection (Figure 5a). We found that H2A.X proteins in the nucleus of the epidermal cells around the hair follicles in the infected organoids were phosphorylated, indicating that SARS‐CoV‐2 infection could lead to double‐strand breaks (DSB) of the epidermal cells around the hair follicles (Figure 5d). Further, results also showed that a redox sensitive transcription factor (NRF2) were activated in the epidermal cells with DNA damage, suggesting that DSB could be caused by oxidative stress after SARS‐CoV‐2 infection (Figure 5d). These results indicate hair follicles could be the target domain of SARS‐CoV‐2 in the COVID‐19 skin, which could lead to the polarity and function of epidermal cells around hair follicles, possibly by affecting DNA damage and oxidative stress of the epidermal cells.

### 2.4 SARS‐CoV‐2 Can Infect the Nervous System of Skin

**Figure 6:**
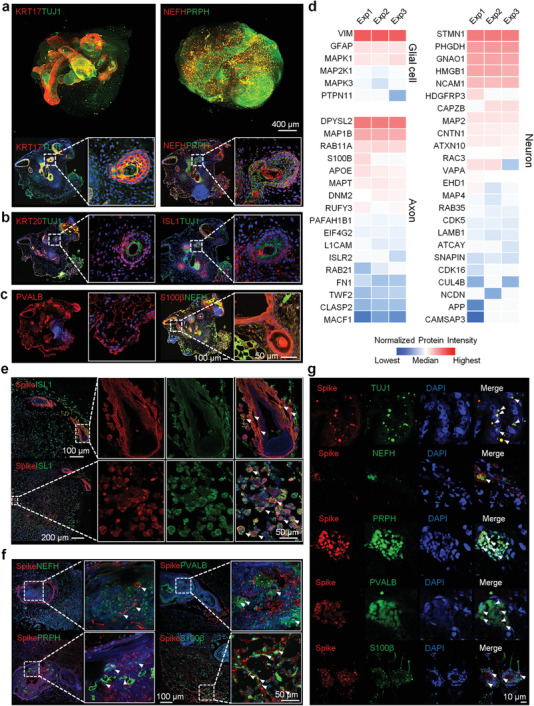
SARS‐CoV‐2 infects the nervous system of the skin. Immunofluorescence of a nervous marker with different cell types: a) KRT17, TUJ1 (pan‐neuronal marker), NEFH (neurofilament heavy chain), and PRPH (peripherin), b) KRT20, TUJ1, and ISL1, and c) PVALB, S100*β*, and NEFH in the normal skin organoids (Scale bar: 400 and 50 µm). d) Heatmap analysis of the proteins identified in the skin organoids that were associated with the development of glial cells, axons, and neurons. Red and blue boxes indicate proteins with high and low intensities, respectively. Immunofluorescence of e) spike and ISL1, f) spike, NEFH, PRPH, PVALB, and S100*β* in the SARS‐CoV‐2 infected skin organoids (Scale bar: 50µm). g) Immunofluorescence of the spike, TUJ1, NEFH, PRPH, PVALB, and S100*β* in the COVID‐19 skin tissues (Scale bar: 10 µm). Exp1, Exp2, and Exp 3 represent different biological repeat of the proteomics experiment (*n* = 3).

We have found that the function of the nervous system in the COVID‐19 skin tissues was severely affected (Figure 1c). Therefore, we investigated whether SARS‐CoV‐2 could directly infect the nerve cells in the skin. The cultured skin organoids showed the presence of the nervous system, expressing pan‐neuronal marker tubulin *β* class III (TUJ1), especially around the hair follicles (**Figure** **6**a). Neurofilament heavy chain (NEFH), peripherin (PRPH), and insulin gene enhancer protein (ISL1) were found to be expressed in the neuronal network along with S100 Calcium Binding Protein B (S100*β*)+ Schwann‐like cells and parvalbumin (PVALB)+ neuron somas (Figure 6a–c and Video S3, Supporting Information). The proteins associated with the development of glial cells, axons, and neurons were successfully identified in the skin organoids. (Figure 6d). After SARS‐CoV‐2 infection, we found that SARS‐CoV‐2 could target TUJ1+, NEFH+, PRPH+, and PVALB+ neurons (Figure 6e,f and Video S4, Supporting Information). S100*β*+ Schwann‐like cells were also found to be infected by SARS‐CoV‐2 (Figure 6f). Further, the COVID‐19 skin tissues, also showed the TUJ1+, NEFH+, PRPH+, PVALB+, and S100*β*+ nerve cells were infected by SARS‐CoV‐2 (Figure 6g and Figure S4f, Supporting Information). Besides, the downregulated proteins in the COVID‐19 skin tissues were mainly enriched in neuroendocrine cells, microglia, astrocytes, satellite cells, neuron and nervous development (Figure 4j). These results indicate that the nervous system could also be the target of SARS‐CoV‐2 in the COVID‐19 skin.

## 3 Discussion

In tissues with a regenerative capacity, the body needs to mobilize a variety of cell types for tissue repair during the process of wound healing, such as activating quiescent reserve stem cells and stromal cells secreting matrix proteins for tissue remodeling. Skin is the first line of defense against external damage in the human body. It is the first organ exposed to chemical damage (e.g., radiation and ultraviolet rays), physical damage (e.g., acute or chronic wounds), and microbial infection (e.g., bacteria or viruses). The integumentary system consists of the epidermis, dermis, dermal appendages, such as hair follicle, sweat glands, sebaceous glands, and nerve cell endings. The interaction of different cell types from epidermis and dermis promotes skin self‐renewal and regeneration during skin homeostasis and injury. For example, the basement membrane from the dermis can maintain the cell fate and function of epidermal stem cells (EpSCs)^[^ ^30^ ^]^ during wound healing. The re‐epithelialization process requires cytokines, inflammatory factors, and ECM secreted by various types of cells in the dermis to promote the proliferation and migration of EpSCs.^[^ ^31^, ^32^ ^]^ In the healing phase of ECM remodeling, the enzyme system secreted by epidermal cells promotes the degradation and assembly of dermal matrix.^[^ ^30^ ^]^ Therefore, to investigate the mechanism of skin injury, we need to establish a relatively ideal model composed of a variety of cells in in vitro culture system, to find a potential therapeutic method for skin repair.

Here, we constructed a skin organoid model induced by hiPSCs and proved that it can respond well to the microbial damage. Through this model, we first assessed that SARS‐CoV‐2 can attack hair follicles directly, which could be related to the hair loss in COVID‐19 patients reported previously.^[^ ^7^ ^]^ HFSCs maintain homeostasis partly from the stem cells in the basal layer (EpSCs) in the skin.^[^ ^33^ ^]^ We found that the proteins associated with the development of EpSCs were acutely downregulated both in the skin tissues from COVID‐19 patients and SARS‐CoV‐2 infected organoids. In addition, we also found the components of BM, an important niche of EpSCs, downregulated, which is consistent with the results from lungs^[^ ^3^ ^]^ and liver.^[^ ^2^ ^]^ This might also be one of the reasons for skin blistering in the COVID‐19 patients. Results also showed that the proliferation ability of the epidermal cells around hair follicles infected by SARS‐CoV‐2 decreased, probably through SARS‐CoV‐2 infection leading to the DSB and oxidative stress, which is consistent with the our previously work.^[^ ^2^, ^3^ ^]^ It has also been reported that some virus can attack the hair follicles, for example, the varicella zoster virus (VZV), a highly human‐specific virus. Studies showed that hair follicles is the initial site of VZV replication in skin.^[^ ^34^ ^]^ There were clinical cases reported the relation of the localized hair loss with VZV infection,^[^ ^35^ ^]^ which is similar with our findings that SAR‐CoV‐2 infection may cause hair loss. Since the use of 3D skin organoid models for studying the pathological mechanisms in SARS‐CoV‐2 infection has not been reported so far, it will not be possible to compare our findings with other skin organoids infected by the virus. Further, whether COVID‐19 can directly attack HFSCs and lead to hair follicle necrosis requires further investigation.

We also found that SARS‐CoV‐2 can attack the neurons in the skin, which is consistent with the previous reports that SARS‐CoV‐2 was able to directly attack the nerve cells in the brain organoids.^[^ ^24^ ^]^ It has been previously reported that mediators secreted by the nervous system play an important role in hair follicle's growth.^[^ ^36^ ^]^ Our results also suggest a possible correlation between the somatic nervous system and hair follicle development. Several studies have found that the herpes simplex virus type 1 can infect skin neurons, which could result in release of cytokines and neuropeptides and affect the neural function and immune system.^[^ ^37^ ^]^ This suggests that SARS‐CoV‐2 infection of the skin nervous system may lead to the disorder of the central system and immune system. In addition, we also found a considerable depletion of proteins related to vascular development and regulation of blood circulation, which may be caused by abnormal blood coagulation of COVID‐19.^[^ ^2^, ^3^ ^]^ Vascular development and hair follicle development are also inseparable. Therefore, there are many direct and indirect factors leading to hair loss in COVID‐19 patients, which need to be explored further. In conclusion, our research suggests that COVID‐19 can directly affect the hair follicles and nerves in the skin. The infection model that we established provides a guideline for subsequent research on the mechanism of SARS‐CoV‐2 infection and drug screening in COVID‐19 patients with affected skin.

Moreover, the skin organoid constructed in this study includes both the surface (epidermis and dermis) and deep (cartilage and subcutaneous fat) structures of skin, which could make it an ideal model for other skin disease research. For example, the patients with scleroderma have high skin fibrosis.^[^ ^38^ ^]^ In addition to dermal vascular malformation and epidermal appendage atrophy, they also show the phenotype of articular cartilage damage and thin adipose layer. For the disease with such complex clinical phenotypes, it could be a good application to treat it using the skin organoids with complete tissue structure and cell types.

## 4 Experimental Section

### Human Subjects

Five COVID‐19 skin samples and six control (non‐COVID‐19) skin samples of the chest were obtained by autopsy from Wuhan Jinyintan Hospital, Wuhan, China (Table S3, Supporting Information). The study was approved by the ethics committees at Wuhan Jinyintan Hospital Ethics Committee (permission number: KY‐2020‐15.01) and Tongji medical school, Huazhong University of Science and Technology (permission number:[2021]IEC‐A001). Written informed consent was obtained from the patients’ families with the permission for publication of all the research results. Laboratory confirmation of SARS‐CoV‐2 infection was performed at Jinyintan and Wuhan Central Hospital and Wuhan Institute of Virology, Wuhan, China. SARS‐CoV‐2 viral RNA was confirmed by real‐time quantitative RT‐PCR or pathological staining.

### Sample Preparation

Samples from the skin tissue and skin organoids were scraped into a new eppendorf tube with 20 µL of urea buffer (8 m urea, 150 × 10^−3^ m Tris‐HCl, 10 × 10^−3^ m dithiothreitol, pH 8.0). An additional 10 µL of buffer was added to the tube. Steel balls were added to the 30 µL of buffer for vibration (70 Hz) for 1 min. After centrifugation at 14 000 × *g* for 10 min at 4 °C, the supernatants were transferred to clean tubes. Next, the extracted proteins were reduced at 37 °C for 1 h and alkylated in 25 × 10^−3^ m iodoacetamide at room temperature for 30 min in the dark. The protein samples were digested with Lys C (1 µg at 37 °C for 4 h) and trypsin (enzyme‐to‐substrate ratio of 1:50 at 37 °C for 16 h), desalted through C18 cartridges (Beijing Qinglian Biotech, China) and vacuum‐dried using a Speed Vac.

### Peptide Pre‐Fractionation using High‐pH High Performance Liquid Chromatography (HPLC)

Pooled peptides were fractioned using high‐pH HPLC to reduce sample complexity. Briefly, the peptides were dissolved in buffer A (2% acetonitrile[ACN], pH 9.5), loaded on an Xbridge C18 column (Waters, MA, USA; 4.6 mm × 100 mm, 130 A°, 5 µm), and eluted with a 70‐min gradient from 0% to 95% of buffer B (98% ACN, pH 9.5) at a flow rate of 0.7 mL min^−1^. Aliquots were combined into 24 fractions before mass spectrometry (MS) analysis.

### Liquid Chromatography‐MS/MS

Samples were measured using an EASY‐nLC 1200 (Thermo Fisher Scientific, Waltham, MA, USA) coupled to a Q Exactive HF‐X Orbitrap mass spectrometer (Thermo Fisher Scientific) via a nano‐electrospray ion source (Thermo Fisher Scientific). Purified peptides were re‐dissolved in mobile phase A (20% ACN and 0.1% formic acid) and directly loaded onto a C18 nano‐capillary analytical column (Beijing Qinglian Biotech, China, 150 µm × 150 mm, 100 A°, 1.9 µm). For the proteome profiling samples, the peptides were separated on an analytical column over a 90 min gradient (buffer A: 0.1% formic acid and 80% H_2_O; buffer B: 0.1% formic acid and 20% ACN) at a constant flow rate of 0.6 µL min^−1^ (0–15 min, 8–12% buffer B; 15–65 min, 12–30% buffer B; 65–80 min, 30–40% buffer B; and 81–90 min, 95% buffer B).

The data‐independent acquisition (DIA) scan mode was used for skin tissue samples. The fractionated samples of the pool were acquired using the top 40 data‐dependent acquisition (DDA) scan mode. Both acquisition schemes were combined with the same liquid chromatography gradient. The mass spectrometer was operated using Xcalibur software (Thermo Fisher). The DDA scan settings on the full MS level included an ion target value of 3 × 10^6^ charges in the 350–1500 *m*/*z* range, with a maximum injection time of 80 ms and a resolution of 120 000 at 200 *m*/*z*. At the MS/MS level, the target value was 5 × 10^4^ charges with a maximum injection time of 45 ms and a resolution of 15 000 at *m*/*z* 200. For MS/MS events only, precursor ions with two to seven charges that were not on the 16 s dynamic exclusion list were isolated within a 1.6 *m*/*z* window. Fragmentation was performed by higher‐energy C‐trap dissociation with a normalized collision energy of 27 eV. DIA was performed with one full MS event, followed by 42 MS/MS windows in one cycle. The full MS settings included an ion target value of 3 × 10^6^ charges in the 350–1500 *m*/*z* range, with a maximum injection time of 50 ms and a resolution of 60 000 at *m*/*z* 200. DIA precursor windows ranged from 378 *m*/*z* (lower boundary of the first window) to 1345 *m/z* (upper boundary of the 42nd window). MS/MS settings included an ion target value of 1 × 10^6^ charges for the precursor window, with an Xcalibur‐automated maximum injection time and a resolution of 30 000 at *m/z* 200.

The label free mass spectrometry technology was applied for both normal and SARS‐CoV‐2 infected skin organoids analysis. Mobile phases A (100% water, 0.1% formic acid) and B solution (100% acetonitrile, 0.1% formic acid) were prepared. The lyophilized powder was dissolved in 10 µL of solution A, centrifuged at 14 000 g for 20 min at 4 °C, and 1 µg of the supernatant was injected into a C18 column. Peptides were separated in an analytical column, using a linear gradient elution. The separated peptides were analyzed by a Q Exactive HF‐X mass spectrometer (Thermo Fisher), with an ion source of Nanospray Flex (ESI), a spray voltage of 2.4 kV and an ion transport capillary temperature of 275 °C. Full scan range was from *m/z* 350 to 1500 with resolution of 120 000 (at *m/z* 200), an automatic gain control (AGC) target value was 3 × 10^6^ and a maximum ion injection time was 80 ms. The top 40 precursors of the highest abundant in the full scan were selected and fragmented by higher energy collisional dissociation and analyzed in MS/MS, where resolution was 15 000 (at *m/z* 200). The AGC target value was 5 × 10^4^. The maximum ion injection time was 45 ms. A normalized collision energy was set as 27%. The intensity threshold was 3.3 × 10^4^, and the dynamic exclusion parameter was 15 s.

### Proteomic MS/MS Data Processing

The MS data of the fractionated pool (DDA MS data) and the single‐shot samples (DIA MS data) were used to generate a DDA library and direct‐DIA library, respectively, which were computationally merged into a hybrid library in Spectronaut (version 14.9.201124.47784, Biognosys, Switzerland). Then, the raw DIA data were processed on Spectronaut using the default settings. All searches were performed against the human UniProt reference proteome sequences, with 20 279 entries downloaded in February 2021. The searches used carbamidomethylation as the fixed modification and acetylation of the protein N‐terminus and oxidation of methionines as the variable modifications. Default settings were used for other parameters. In brief, a trypsin/P proteolytic cleavage rule was used, permitting a maximum of two miscleavages and a peptide length of 7–52 amino acids. Protein intensities were normalized using the “Local Normalization” algorithm in Spectronaut based on a local regression model; the retention time prediction type was set to dynamic iRT and correction factor for a window. The mass calibration was set to Local Mass Calibration. Decoy generation was set to Inverse. Interference correction on the MS2 level was enabled, removing fragments for quantification based on the presence of interfering signals, but maintaining at least three fragments for quantification. The false discovery rate was estimated using the mProphet approach and set to 1% at the peptide level.

The raw files of label free experiment for skin organoids were analyzed using the MaxQuant software (version 1.6.5.0).^[^ ^39^ ^]^ Proteins were identified by searching against a database containing the SwissProt human sequences (accessed on June, 2021, containing 20 395 human proteins) with the common contaminants included in MaxQuant. Peptides were identified using a precursor mass tolerance of 4.5 ppm and a fragment mass tolerance of 20 ppm. Cysteine carbamidomethylation was set as the fixed modification, and N‐terminal acetylation and methionine oxidation served as variable modifications. Two missed cleavages were allowed, and trypsin was set as the reference enzyme. Automatic target and reverse database searches were enabled with a maximum false discovery rate of 0.01 for peptide and protein identification. Protein quantification was performed according to the intensity‐based absolute quantification method iBAQ^[^ ^40^ ^]^ as implemented in MaxQuant.

### The Culture of Skin Organoids

The human induced pluripotent stem cells (Human iPSC Line nciPS02, RC01001‐B, Female, Nuwacell Biotechnologies Co., Ltd) were cultured on the 6‐well plate, coated with 1% Matrigel (354277, BD, USA). For differentiation, hiPSCs were seeded in a U‐bottom low‐attachment 96‐well plates (Corning, 7001) at a concentration of 3500 cells per well, and maintained in the Essential 8 medium supplemented with 20 × 10^−6^ m Y‐27632 (E8‐20Y) in an incubator for 24 h. Then, 100 µL fresh E8 medium without Y‐27632 were added into each well and then continue to be incubated for 24 h. Next, all cell aggregates were transferred to a new U‐bottom low‐attachment 96‐well plate in 100 µl of Essential 6 medium containing 2% Matrigel (Corning), 10 × 10^−6^ m SB431542 (R&D), 4 ng mL^−1^ FGF (R&D) and 2.5 ng mL^−1^ BMP4 (R&D), defined as E6SFB, and incubated in 37 °C incubator with 5.0% CO_2_. On day 3 of differentiation, 200 ng mL^−1^ LDN‐193189 (BMP inhibitor, R&D) and 50 µg mL^−1^ FGF were added to induce CNC cell formation. On day 6 of differentiation, fresh E6 medium was added, bringing the final volume to 200 µL. On day 12, all aggregates were transferred into 24‐well low‐attachment plates (Corning) with organoid maturation medium (OMM) containing 1% Matrigel (OMM1%M). The OMM contains advanced Dulbecco's modified Eagle medium: nutrient mixture F‐12 (Advanced DMEM/F‐12; Gibco) and neurobasal medium (Gibco) at a 1:1 ratio, 1× GlutaMax (Gibco), 0.5× B‐27 minus vitamin A (Gibco) and 0.5× N‐2 (Gibco) supplements, 0.1 × 10^−3^ m 2‐mercaptoethanol (Gibco) and 100 µg mL^−1^ normocin. On day 15, 250 µL medium were removed from 24‐well plates, and 250 µL fresh OMM1%M were added in the plates. And from day 15 or more, half of the medium was replaced every three days or every other day according to the growth status of organoids. On day 75, TGFBI (Transforming Growth Factor‐Beta‐Induced Protein Ig‐H3) was added in the culture medium to promote epidermal development.

### H&E Staining and Immunofluorescence

Samples were fixed in 4% formaldehyde overnight at 4 °C and then processed through a dehydration gradient. After the tissues were embedded in paraffin, they were cut into 4 µm thick sections for H&E staining and immunofluorescence analysis. For immunohistochemical staining, the sections were deparaffinized and heated in a microwave to a boil for at least 12 min with antigen retrieval buffer and then removed by endogenous catalase in 0.3% H_2_O_2_ for 30 min after cooling. The sections were then blocked with normal horse serum in Tris‐buffered saline for 1 h, blocked with Avidin/Biotin Blocking Kit, and stained with antibodies overnight at 4 °C. The sections were then stained with a secondary antibody. For immunofluorescence staining, the cells were fixed in 4% formaldehyde for 20 min and then washed in phosphate buffer saline. Then, the cells were treated with 0.25% Triton X‐100 for 20 min to remove plasma membranes, blocked in 10% serum for 1 h at room temperature (RT), and incubated with primary antibodies overnight at 4 °C. After incubation for 1 h at RT with secondary antibodies and counterstaining with 4′,6‐diamidino‐2‐phenylindole (DAPI), sections were sealed with Fluoro‐Gel for Photography. Negative control samples were incubated with secondary antibody alone. 2D images were taken at 10 × 20 × /40× /60× /100× magnification and analyzed using Volocity Demo (×64). 3D images and videos were taken at light‐sheet multiview selective‐plane illumination microscope with 0.87 × 0.87 × 1 µm voxel size, and 1.8 × 1.8 × 2 mm^3^ shooting field of view.

*Transmission Electronic Microscopy*: After inactivating in 2.5% (v/v) glutaraldehyde for at least 24 h at 4℃, a small fraction (<1 mm^3^) of freshly dissected skin tissues were fixed in 1% (w/v) osmic acid for 2 h at 20℃. Then, samples were dehydrated and infiltrated. The ultrathin sections (80–100 nm) were obtainedand further stained with 2% uranyl acetate and lead citrate. The samples were examined by a HITACHI H‐7000FA transmission electron microscopy at an accelerating voltage of 200 kV.

### SARS‐CoV‐2 Infection Experiment

For viral infection, 140‐day‐old human skin organoids were transferred into 12‐well plates. Each well contained one organoid in 1 mL OMM medium and then incubated with 1 × 10 5 TCID50 of SARS‐CoV‐2 at 37 °C. The half of the medium was removed and replaced with 500 µL fresh medium at 48 h post‐infection. At 96 h post‐infection, the infected organoids were taken and subjected for fixation in 10% formaldehyde for one week.

### Statistical Analysis

Statistical analyses were performed using R package (version 4.0.3) and Python package (version 3.6.0). For skin tissues, COVID‐19 samples (*n* = 5) and healthy control samples (*n* = 6) was used for proteomics and validation experiments. For skin organoids, each experiment was performed three times (*n* = 3) both for the normal control and SAR‐CoV‐2 infencted samples. The quantification values of the identified proteins were normalized by taking the fraction of the total, followed by multiplication by 10^6^ and log2 transformation, and no outliers were removed in this study. Pairwise comparisons were used to identify proteins whose expression was significantly different between COVID‐19 patients with infection and the controls. A moderated t‐statistic for each protein was performed using the R package Limma (version 3.46.0).^[^ ^41^ ^]^ The Benjamini–Hochberg adjusted *p*‐value was <0.01 were considered statistically significant. The proteins that met the fold‐change threshold were taken as upregulated (log_2_ COVID‐19/Control >1 or log_2_ Infected/Control >1) or downregulated (log_2_ COVID‐19/Control < −1 or log_2_ Infected/Control < −1) where it indicated. The protein identification results are shown as means ± standard deviation (Table S1, Supporting Information). The database for annotation, visualization and integrated discovery (DAVID, https://david.ncifcrf.gov/)^[^ ^42^ ^]^ was used to annotate the proteins according to biological processes and cellular components via Gene Ontology^[^ ^43^ ^]^ analysis. The cell type signature gene sets were downloaded from the Molecular Signatures Database (MSigDB),^[^ ^44^ ^]^ and the identified proteins were annotated and calculated the enrichment p‐value using the Python package scipy (version 1.2.0). The PCA of the proteins whose values in each sample were valid was performed using the R package ape (version 5.4.1). The protein‐protein interactions were retrieved from the search tool for recurring instances of neighbouring genes database (STRING, https://string‐db.org/),^[^ ^45^ ^]^ and the network was built using Cytoscape (version 3.8.2).^[^ ^46^ ^]^ The Matrisome database^[^ ^47^ ^]^ (http://matrisomeproject.mit.edu/) was used to annotate the ECM and to define the six categories of core ECM (including collagens, proteoglycans and ECM glycoproteins) and ECM‐associated proteins (including ECM regulators, ECM‐affiliated proteins and secreted factors). The circlize package^[^ ^48^ ^]^ (version 0.4.11) was used to circularly visualize the expression correlation of proteins identified in skin organoids with the different cell types.

## Conflict of Interest

The authors declare no conflict of interest.

## Author Contributions

L.L. and J.M. conceived the overall study and designed experiments. L.L., M.W., Y.Z., and S.Z. had full access to all the data in the study and took responsibility for the accuracy of the data analysis. L.L., D.G., Q.Z., and L.Lv contributed to the culture of skin organoids. Y.Z., Q.L., and L.M. prepared the skin tissues from COVID‐19 patients and control individuals. M.W., J.L., Z.H., H.H., and Y.L. performed viral infection experiments. J.M., L.L., X.L., and Y.Z. performed proteomics experiments and bioinformatics analysis. L.L., D.G., L.Lv, Y.W., and Z.W. performed most of pathological staining experiments. L.L., J.M., J.L., Y.Z., and M.W. wrote and edited the paper. S.Z., Y.Z., Z.W., and Z.H. provided funding support. All authors made important comments to the paper.

## Supporting information

Supporting Information

Supplemental Video 1

Supplemental Video 2

Supplemental Video 3

Supplemental Video 4

Supplemental Table 1

## Data Availability

The data that support the findings of this study are openly available in ProteomeXchange Consortium at http://proteomecentral.proteomexchange.org/, reference number 28058.
