# Supplementary material for: Establishment of Human Pluripotent Stem Cell‐Derived Skin Organoids Enabled Pathophysiological Model of SARS‐CoV‐2 Infection
Source: Adv Sci (Weinh). 2021 Dec 31;9(7):2104192. doi: 10.1002/advs.202104192 (PMC8895131; doi:10.1002/advs.202104192)
Supplement: Supplementary file 1 — Supporting Information [file ADVS-9-2104192-s002.pdf]

© 2020 Wiley-VCH GmbH

ADVANCED SCIENCE
Open Access

Supporting Information

for *Adv. Sci.,* DOI: 10.1002/advs.202104192

Establishment of human pluripotent stem cell-derived skin organoids enabled pathophysiological model of SARS-CoV-2 infection

Jie Ma, Jia liu, Dunqin Gao, Xiao Li, Qiyu Zhang, Luye Lv, Yujie Wang, Jun Li, Yunping Zhu, Zhihong Wu, Hengrui Hu, Yufeng Li, LongDa Ma, Qian Liu, Zhihong Hu, Shuyang Zhang, Yiwu Zhou*, Manli Wang*, and Ling leng**

Supporting Information

Establishment of human pluripotent stem cell-derived skin organoids enabled pathophysiological model of SARS-CoV-2 infection

Jie Ma†, Jia liu†, Dunqin Gao†, Xiao Li†, Qiyu Zhang, Luye Lv, Yujie Wang, Jun Li, Yunping Zhu, Zhihong Wu, Hengrui Hu, Yufeng Li, LongDa Ma, Qian Liu, Zhihong Hu, Shuyang Zhang, Yiwu Zhou*, Manli Wang*, Ling leng**

† These authors contributed equally to this work.

* Correspondence: Ling Leng (lengling@pumch.cn) or Manli Wang (wangml@wh.iov.cn) or Yiwu Zhou (zhouyiwu@hust.edu.cn) or Shuyang Zhang (shuyangzhang103@nrdrs.org).

Supplementary Figures

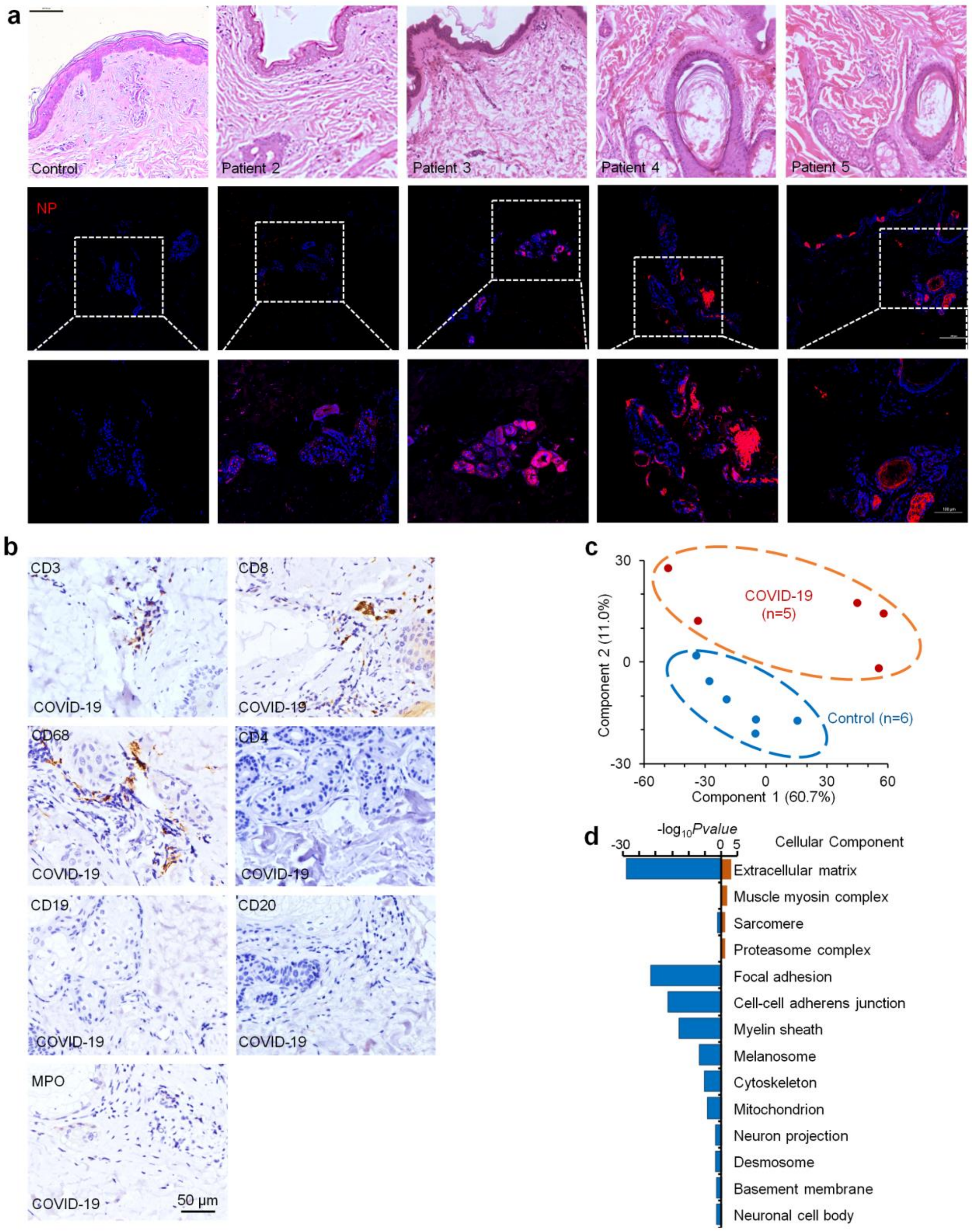

Figure S1. Pathological features of COVID-19 skin tissues. a) H&E and NP protein staining of SARS-CoV-2 infected and control skin tissues (Scale bar: 100 μm). b) Immunohistochemical staining of T cell markers (CD3, CD8 and CD4), macrophage marker (CD68), B cell markers (CD19 and CD20), and neutrophil marker (MPO). schematic representation of the experimental workflow of the skin organoids culture,

SARS-CoV-2 infection, quantitative proteomic, bioinformatics analysis, and biological validation. c) PCA analysis of the proteome profiles of all proteins from COVID-19 and control skin tissues. Biological repeats produced for each skin sample are represented by different colored points. d) Biological process analyses of the upregulated and downregulated-expressed proteins between COVID-19 (n=5) and control (n=6) skin tissues. Upregulated-expressed and downregulated-expressed proteins: t-test, Benjamini–Hochberg (BH) adjusted *p*-value < 0.01 and $\log_2$COVID-19/Control > 1, and BH adjusted *p*-value < 0.01 and $\log_2$COVID-19/Control<-1.

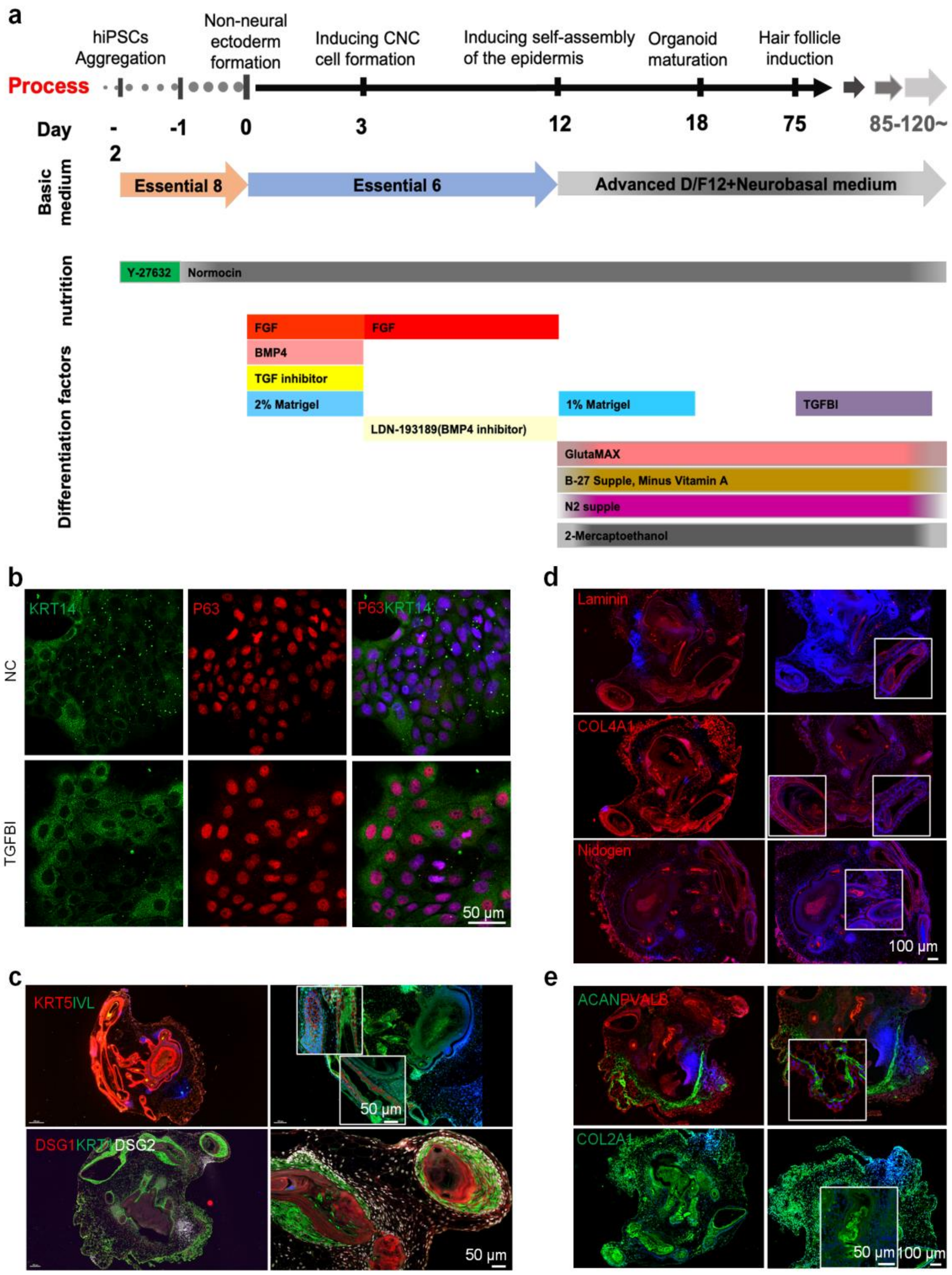

Figure S2. Formation of skin organoids *in vitro*. a) Skin organoid differentiation protocol. Immunofluorescence of KRT14 and P63 in the primary EpSCs with TGFBI treatment (100 ng/mL) for 48 h (b), the epidermal basal cell markers (KRT5), mature epithelium markers (KRT1 and IVL), and desmosomes (DSG1 and DSG2) (c), basement membrane components (laminin, COL4A1, and nidogen) (d), dermal components (ACAN and COL2A1) in the skin organoids (e) (Scale bar: 50 and 100 μm).

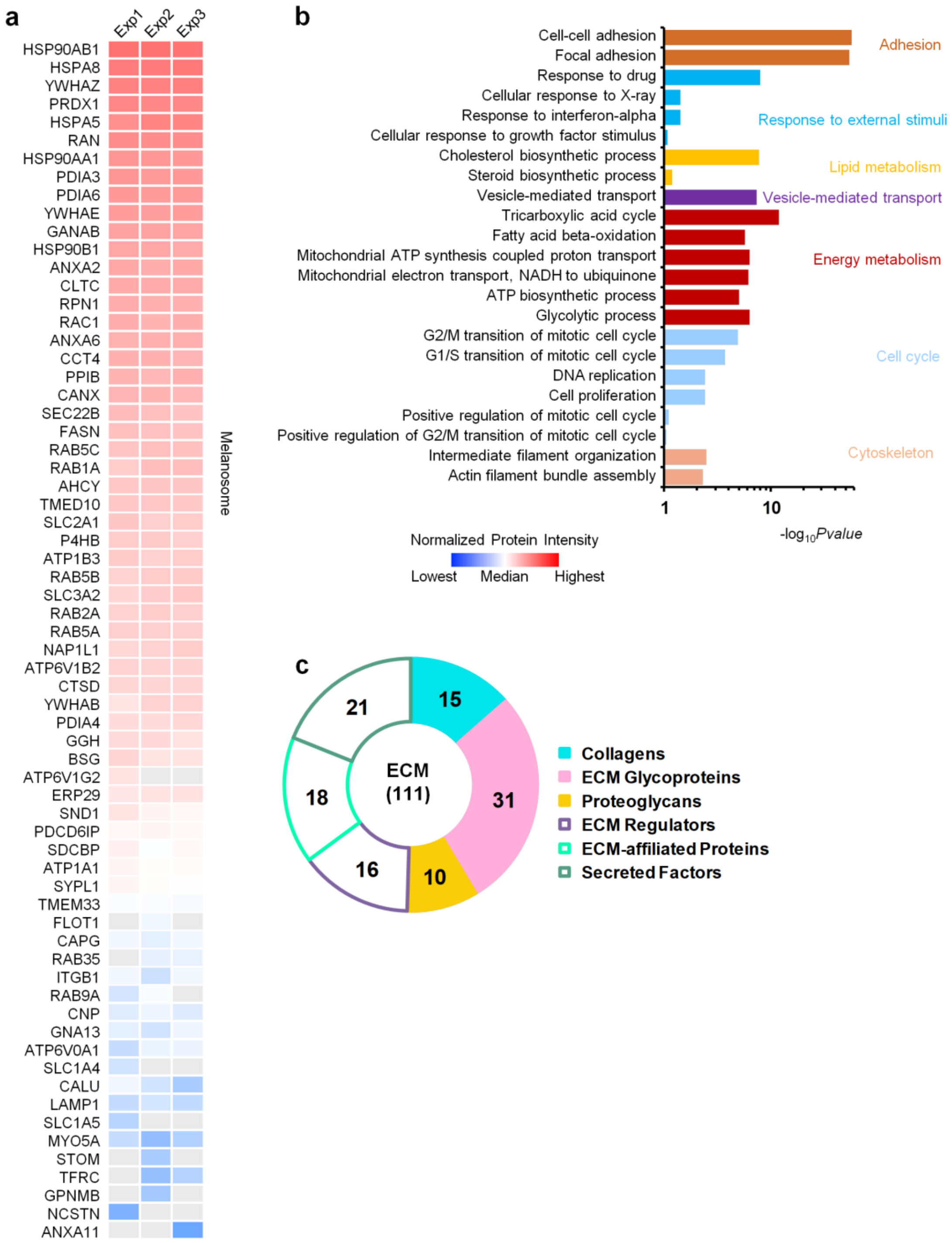

Figure S3. Functional analysis of proteins identified in the skin organoids. a) Heatmap analysis of the proteins identified in the skin organoids that were associated with melanosomes. Red and blue boxes indicate proteins with high and low intensities, respectively. b) Biological process analysis of proteins identified in the skin organoids. c) Pie charts represent the composition of six components' numbers of the ECM

proteins of the skin organoids. Cyan, pink, and yellow circles correspond to collagens, ECM glycoproteins, and PGs, respectively; while blue, green, and atrovirens circles correspond to ECM regulators, ECM-affiliated proteins, and secreted factors, respectively. Exp1, Exp2, and Exp 3 represent different biological repeat of the proteomics experiment (n=3).

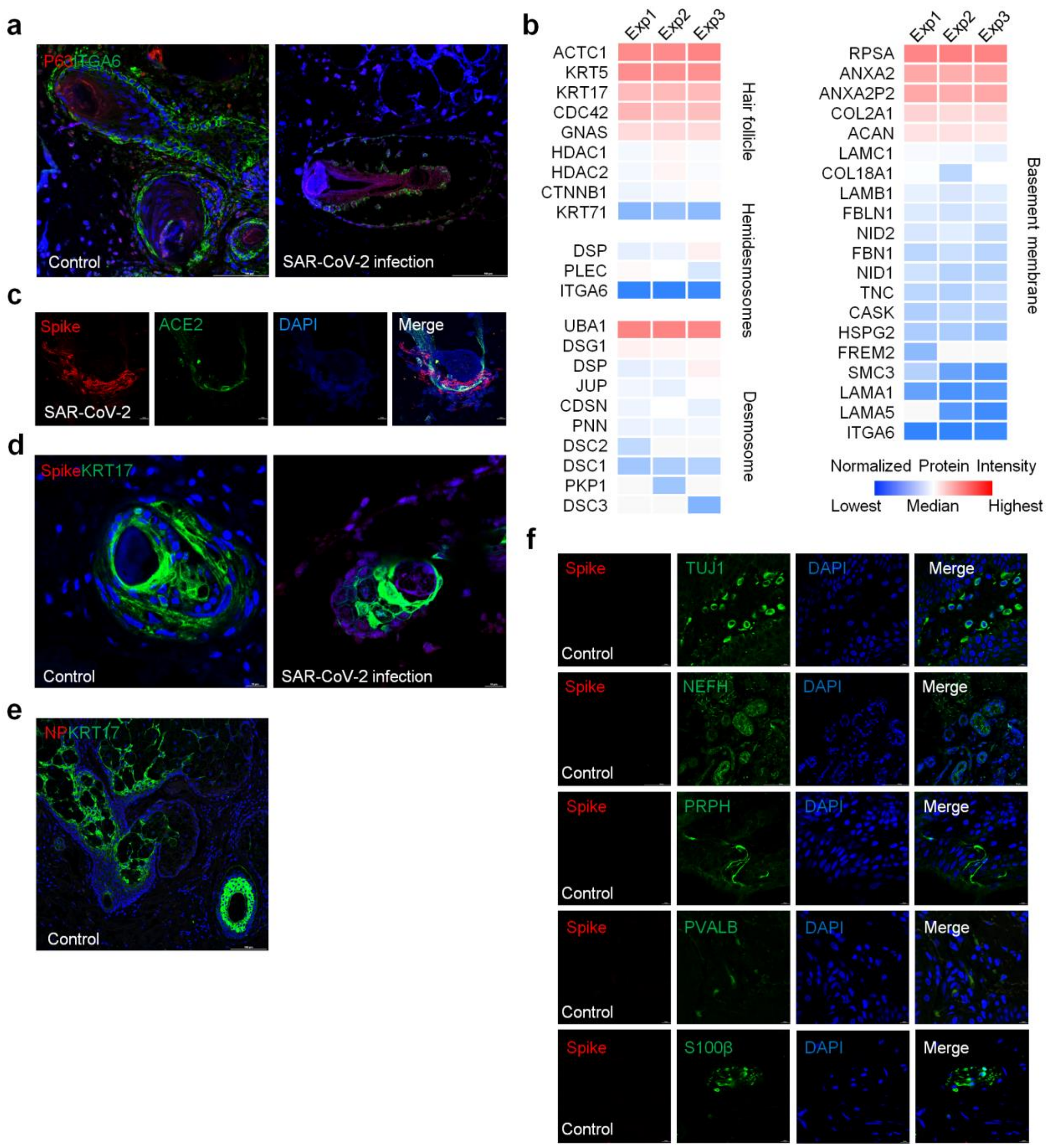

Figure S4. Characteristic of COVID-19 skin tissues. Immunochemical staining of P63 and ITGA6 in the SARS-CoV-2 infected skin organoids (Scale bar: 100 μm) (a). b) Functional analysis of proteins identified in the SARS-CoV-2-infected skin organoids. Immunofluorescence staining of spike and ACE2 (Scale bar: 10 μm) (c), spike and KRT17 (Scale bar: 10 μm) (d) in the SARS-CoV-2 infected and control skin organoids, NP and KRT17 (Scale bar: 10 μm) (e), spike, TUJ1, NEFH, PRPH, PVALB, and S100β in the control skin tissues (Scale bar: 10 μm) (f). Exp1, Exp2, and Exp 3 represent different biological repeat of the proteomics experiment (n=3).

Legends for Supplementary Table S1 to S5

Table S1. All protein identified in normal and SARS-CoV-2 infected skin organoids.

Table S2. All ECM proteins identified in normal skin organoids.

Table S3. Overview of the characteristics of patients diagnosed with COVID-19 and healthy donors involved in the study.

Table S4. Companies providing equipment, reagents and/or supplies.

Table S5. List of all abbreviations of proteins and genes described in this study.

Legends for Supplementary Videos S1 to S4

Video S1. The hair follicle of normal skin organoid (KRT17+ (red), TUJ1+ (green)).

Video S2. SARS-CoV-2 can infect the hair follicle of skin organoid (KRT17+ (green), NP+ (red)).

Video S3. The nervous system of normal skin organoid (NEFH+ (red), PRPH+ (green)).

Video S4. SARS-CoV-2 can infect the nervous system of skin organoid (TUJ1+ (green), Spike+ (red)).
